# Experiences of stroke survivors and measurement of post stroke participation and activity across seasons—A mixed methods approach

**DOI:** 10.1371/journal.pone.0259307

**Published:** 2021-10-29

**Authors:** Ruth Barclay, Leanne Leclair, Sandra C. Webber

**Affiliations:** 1 Rady Faculty of Health Sciences, Department of Physical Therapy, College of Rehabilitation Sciences, University of Manitoba, Winnipeg, Canada; 2 Rady Faculty of Health Sciences, Department of Occupational Therapy, College of Rehabilitation Sciences, University of Manitoba, Winnipeg, Canada; Ehime University Graduate School of Medicine, JAPAN

## Abstract

Participation and activity post stroke can be limited due to adverse weather conditions. This study aimed to: Quantify and compare summer and winter participation and activity, and explore how community dwelling people with stroke describe their feelings about their level of participation and activity by season. This embedded mixed-methods observational study took place in a city with weather extremes. Community dwelling individuals at least one year post-stroke, able to walk ≥50 metres +/- a walking aide were included. Evaluations and interviews occurred at participants’ homes in two seasons: Reintegration to Normal living Index (RNL), Activities-specific Balance Confidence (ABC) and descriptive outcomes. Participants wore activity monitors for one week each season. Analysis included descriptive statistics, non-parametric tests and an inductive approach to content analysis. Thirteen individuals participated in quantitative evaluation with eight interviewed. Mean age 61.5 years, 62% female and mean 6.2 years post-stroke. No differences between winter-summer values of RNL, ABC, or activity monitor outcomes. However, participants felt they could do more and were more independent in summer. The winter conditions such as ice, snow, cold and wind restricted participation and limited activities. Nonetheless, many participants were active and participated despite the winter challenges by finding other ways to be active, and relying on social supports and personal motivation. The qualitative findings explained unexpected quantitative results. Participants described many challenges with winter weather, but also ways they had discovered to participate and be active despite these challenges. Changes to future studies into seasonal differences are suggested.

## Introduction

Worldwide, it is estimated that there are more than 80 million people who are living with the effects of a stroke [1]. An estimated 741,000 Canadians are living with stroke [2]. People with stroke who are living in the community may be experiencing participation restrictions (for example, challenges with taking part in social activities) and activity limitations (for example, difficulty walking or moving about) [3]. Participation is defined in the World Health Organization International Classification of Functioning (ICF) as “involvement in a life situation” [4]. Participation is known to be decreased in people with stroke [5].

Activity is defined as the “execution of a task or action by an individual” [4]. Examples of activity include walking and activities of daily living. Community dwelling people with stroke are generally less physically active, take fewer steps per day, and walk slower (mean 0.6 m/s) than age-matched controls [6]. Increased activity limitations over time post stroke have been associated with depression [7], while participation in meaningful activities is associated with better emotional well-being [8]. Individuals post stroke with poor walking endurance,poor social support and with potential depression demonstrated poor participation [9]. After stroke, decreased community ambulation and social participation have been shown to be associated with decreased walking endurance, while people with stroke are less likely to walk outdoors in cold weather months [10]. In a Japanese study, falls were more frequent in winter than in other seasons, both indoors and outdoors [11]. The study of barriers to activity and participation levels in stroke survivors in different seasons is therefore an important area of study.

Environmental factors can act as either barriers or facilitators to participation and activity [12]. The natural environment aspects of climate, including temperature, precipitation and seasonal variation [4] can affect the participation and activity of people with stroke. Extreme weather has been shown to be a barrier for people with spinal cord injury, traumatic brain injury, and stroke [13]. Weather conditions and temperature have been identified as environmental level barriers by people with stroke by, for example, influencing transportation to social gatherings [14]. Stroke survivors have described avoiding rain, snow, ice and wind as well as preferring to go out during the summer as opposed to the winter [15]. Weather conditions affect the ability of community dwelling stroke survivors to walk in their community, thereby affecting both activity and participation [16].

In a study of older adults who lived ‘mid-latitude’ (average mean difference of temperature summer to winter ~ 23° C, N 36° 43.2’), steps per day were significantly decreased in winter compared to summer [17]. Winnipeg, Manitoba, Canada is at a higher latitude (N 49° 55’) and has a wider mean temperature difference (~36.1° between July and January) [18]. There is snow, typically beginning in November until April, and extreme cold in winter months, with rain and heat in summer months. Evaluating seasonal differences in participation and activity in community dwelling people living with stroke in a city with seasonal weather extremes is important to assist with designing future interventions for safety in winter activity and participation.

### Objectives

1. Quantify and compare summer and winter participation scores, balance confidence, and activity levels (steps/day and characterization of walking bouts) in people with stroke.
2. Explore how community dwelling people with stroke describe their participation and activity in relation to time of year/weather and participants’ feelings about and thoughts on their level of participation in winter and summer.

## Materials and methods

This study is an embedded mixed method observational study with quantitative assessment at two times, each closely followed by the qualitative component for a subset of participants. Qualitative data was used to explain quantitative results. Recruitment was through an email list, newsletter and presentations to members of a local stroke recovery organization, through the city newspaper, through letters to previous outpatients of a university neurorehabilitation clinic, and with posters at the physiotherapy services of a hospital outpatient department for people with neurological conditions.

Ethics approval was received from the University of Manitoba Health Research Ethics Board, HS20174 (H2016:372). All participants signed a written consent form which was explained to them by a research assistant with opportunity to ask questions.

A sample size calculation was completed using the potential difference in participation scores between seasons: with a two-tailed α of 0.05, one-tailed β of 0.20 (80% power) and an estimated medium effect size of 0.50 [19], a sample size of 31 was required [20]. A medium effect size was also expected to be appropriate for comparing step counts across seasons in people with stroke [6,17].

Inclusion criteria were: community dwelling individuals with stroke, aged 45 and over, at least 12 months post stroke, and a self-reported ability to walk independently 50 metres (with or without a walking aid). Exclusion criteria were: unable to answer surveys, self-reported decreased sensation to both lower legs, self-reported regular swelling in both ankles, Mini-Mental State Exam-telephone version score of <18/22 [21], or having been told to restrict physical activity by a physician.

### Outcomes

Participation was evaluated using the Reintegration to Normal Living Index (RNL). Scores of the RNL have shown good reliability and validity in stroke [22,23]. The RNL has 11 questions with three scoring versions: a 10 point visual analogue scale, 3 point scoring, or 4 point scoring [22]. We utilized the 4 point version (from 0 to 3), where a higher score reflects higher participation/reintegration. This measure is a self-perceived outcome–it is completed by the participant. The total score ranges from 0 to 33 [24].

The Activities-specific Balance Confidence Scale (ABC) evaluates balance-related confidence (self-efficacy) with 16 items on a 0–100 scale. Scores have been validated with stroke [25]. A higher score represents higher self-efficacy and is a self-perceived outcome.

Steps per day and characterization of walking bouts were used as objective measures of activity level. The ActiGraph GT3X+ activity monitor (http://www.actigraphcorp.com/products/wgt3x-monitor/, ActiGraph, Pensacola, FL) worn at the ankle has been shown to accurately detect steps in slow walkers who walk at speeds of < 0.8 metres/second (m/s) [26,27]. This device has been shown to be reliable [28] and to have good validity in free living conditions [29]. In older adults who walk slowly, it has been shown to be accurate in detecting steps when worn at the ankle and the low-frequency extension (LFE) algorithm is used in analysis [26,27].

The GT3X+ monitor was attached to the lateral less-affected ankle with an elastic strap [30]. As per the criteria, participants were excluded if they had decreased sensation or regular swelling in both lower extremities to prevent potential problems with wearing an ankle strap. Participants were shown how to adjust the elastic strap for comfort and how to wear it over winter boots or clothing.

#### Outcomes describing the participants

The telephone interview version of the Chedoke-McMaster Stroke Assessment–Activity Inventory(TCMSA-AI), is a measure of activity for use with community dwelling people with stroke, scored on a 14 to 100 scale [31]. Gross motor function (transfers, bed mobility) and walking (indoor, outdoor and stairs) are evaluated. A higher score is interpreted as higher independence in gross motor function and walking activities. This measure is self-perceived and completed by interview.

The Timed Up and Go (TUG) is a measure of mobility and balance, measured by the time in seconds that it takes to stand up from an armchair, walk 3 metres, turn around, walk back and sit down. Reliability and validity has been studied in older adults with numerous health conditions, including stroke [32,33]. This measure was observed in the home of each participant. Two trials were completed. The first trial was considered practice and the second trial was used in analysis. A higher score (in seconds) reveals that it takes longer to complete the task. A score of greater than 14 seconds is suggested as being associated with a higher risk of falls after stroke [34].

The Stroke Impact Scale (SIS) recovery scale is a 0–100 vertical visual analogue scale which asks, “On a scale of 0 to 100, with 100 representing full recovery and 0 representing no recovery, how much have you recovered from your stroke?” [35] Scores of the SIS recovery have shown good reliability and validity post stroke [35]. This item is self-perceived and represents a higher perception of recovery with a higher score.

We collected summary weather information for the day of each assessment from the Environment Canada Historical climate data website [18]. The average temperature for the 7 day period when the participant was wearing the activity monitor was calculated.

### Seasonal assessments

Participants were assessed in their homes at two times: summer, or warmer weather (June 1 to October 10) and winter, or colder weather (October 10 to March 30). Baseline data was collected between Jan 2017- Jan 2019. Some participants started the study in the summer and some in the winter. At each assessment time, the research assistant asked the participant to complete the RNL, SIS recovery, ABC, TCMSA-AI, and perform a standardized TUG. Participants were asked to wear an ActiGraph GT3X+ activity monitor for one week on a strap around their less-affected ankle, during waking hours. They also completed an activity/wear log for the week in which they wore the activity monitor.

### Interviews

Semi-structured interviews to explore reasons for answers to RNL items in relation to time of year/weather and participants’ feelings about and thoughts on their level of participation and activity in winter and summer, occurred with a subset of participants who were interested in being involved in the interviews. The interview occurred within one week after the activity monitor assessments were completed. Interviews were digitally recorded, transcribed verbatim by a transcriptionist, and anonymized.

### Analysis

Due to the slow pace of enrolment, recruitment was stopped after the thirteenth participant. Descriptive statistics were calculated for all outcomes. Paired t-tests were planned for objective 1, however with small sample size and non-normal data, we utilized Wilcoxon Signed Rank Test to estimate any differences between seasons [36]. Mean and median RNL scores, ABC scores, steps per day and characterization of walking bouts were compared between seasons.

The GT3X+ monitors were initialized to collect data at 100Hz and activity data were downloaded using ActiLife, version 6 (ActiGraph LLC, Pensacola, FL). Data were downloaded in 15 second and 60 second epochs (blocks of time) using the LFE filter. Intervals with ≥ 90 consecutive minutes with zero activity counts (allowing up to 2 consecutive minutes of counts 0–100) were marked as non-wear time [37]. Minimum valid wear time was 8 hours a day and participants needed to have ≥ 8 hours of wear time on at least 4 days for their data to be included in analyses. In addition to calculating steps per day, we determined peak 1-minute cadence (highest steps/min during the day), and peak 30-minute cadence (average steps/min in the 30 highest but not necessarily consecutive minutes during the day) [38]. We also determined the number of short (≤ 40 steps), medium (41–299 steps) and long (≥ 300 steps) walking bouts each day [30]. Similar to Roos et al [30], we analyzed data in 15 second epochs and required ≥ 6 steps/epoch for steps to be considered as part of a walking bout. When steps fell below 6 steps/epoch this signified the end of a walking bout.

For objective 2, a qualitative descriptive approach [39] with an inductive approach to content analysis was taken. Throughout the research process issues related to credibility, dependability, confirmability and transferability were addressed [40,41]. Researchers began by immersing themselves in the transcripts in order to identify dimensions or themes that seemed meaningful to the producer of each message. Creswell suggests several steps to engage the researcher in a systematic process of analyzing textual data including: organizing and reading the data; developing a codebook; coding the data; using descriptive wording for coding; performing a preliminary analysis; recoding if necessary; and describing categories for analysis to be represented in the qualitative narrative [42]. Two authors (LL and RB) completed the qualitative data analysis with the assistance of a research assistant. All three evaluated four transcripts each, compared codes and notes and developed a codebook. The research assistant continued doing the coding with regular meetings with RB and LL. Using the codes, LL and RB developed categories and themes. Data from the quantitative and qualitative components were combined by using the qualitative results to help explain the quantitative results.

## Results

Twenty-two people were screened over a period of Jan 2017- Jan 2019. Nine people did not meet the inclusion/exclusion criteria. Two people had regular swelling in the lower limb, five were below the MMSE score of 18, one was not living independently in the community and one was unable to start the study for medical reasons. Thirteen people provided consent and started the study. Six participants started in the winter while seven started in summer. Refer to Table 1 for characteristics of the participants. Of all participants, 84.6% (11/13) completed both summer and winter home assessments; 76.9% (10/13) completed both summer and winter activity monitor assessments. See Table 2. The month and the temperature on the day that each participant had their home-based assessment is summarized in Table 3.

**Table 1:** Participant characteristics at initial assessment.

(n = 13)	Frequency (%) or Mean (SD)	Median (25^th^,75^th^ percentile)	Range
**Sex (F)**	8 (61.5%)	-	-
**Affected side (left, right, both)**	6, 6, 1 (46%, 46%, 8%)	-	-
**Walking aid use (4 wheeled walker)**	3 (23%)	-	-
**Retired**	8 (61.5%)	-	-
**Initial assessment in summer**	6 (46.2%)	-	-
**Age**	61.5 (15.5)	64 (53.5,71.0)	32–79
**Time post stroke (weeks)**	319.7 (334.3) [6.2 years]	166 (120.0,599.5) [3.2 years]	61–877 [1.2–16.9 years]
**Number of Health Conditions**	2.1 (2.6)	2.0 (0.5,3)	0–8
General health* **(1 = excellent, 5 = poor)**	2.7(0.7)	3.0 (2.0,3.0)	1–4
**SIS recovery (0–100)**	70.8 (13.2)	75.0 (60.0,80.0)	50–90
**CMSA-AI Total (14–100)**	80.2 (15.5)	84.0 (65.5,95.0)	50–100
**TUG (seconds, lower score is better)**	21.9 (12.0)	21.5 (9.4,27.4)	7.3–45.7

SD = standard deviation, SIS = Stroke Impact Scale, CMSA-AI = Chedoke-McMaster Stroke Assessment-Activity Inventory, TUG = Timed Up and Go

*“Compared to other persons your age, how would you rate your health?”.

**Table 2:** Summary of participants involved in each aspect of the study.

	Winter assessment	Summer assessment	Both seasons	Total
**Home based outcomes**	11	13	11	13
**Accelerometry**	11	12	10	12
**Qualitative**	6	7	5	8

**Table 3:** Month and temperature of the day each participant completed home based assessment.

Participant	Winter Assessment	Mean temperature (°Celsius)	Summer Assessment	Mean temperature (°Celsius)
2	March	-18.6	July	22.1
3	February	-3.3	June	17.3
4	January	1.3	September	24.6
5	March	2.1	June	17.3
6	March	2.3	July	24.1
7	February	-1.8	July	24.1
11	January	-10.5	October	2.6
14	na	na	June	20.7
18	January	-5.0	June	21.3
19	na	na	June	20.7
20	January	-18.1	July	19.1
22	January	-8.7	June	20.7
23	January	-6.1	August	23.3

na = not assessed.

Table 4 shows the results of the self-perceived measures of the RNL, as a measure of participation, and the ABC as a measure of balance confidence, for those participants who had evaluations in both summer and winter seasons. The median (25^th^,75^th^ percentile) of RNL was 26.0 (19.0,31.0) in winter and 27.0 (20.0,31.0) in summer, with Wilcoxon Signed Rank Test Z = 27.5 (p value 0.55).

**Table 4:** Self-perceived outcomes by season.

	Winter (n = 11)			Summer (n = 11)			Difference	
Measure	Mean (SD)	Median (25^th^,75^th^ percentile)	Range	Mean (SD)	Median (25^th^,75^th^ percentile)	Range	Wilcoxon Signed Rank Test Z	p value
**RNL (0–33)**	25.5 (6.6)	26.0 (19.0,31.0)	13–33	26.1 (5.4)	27.0 (20.0,31.0)	19–33	27.5	0.55
**ABC (0–100)**	72.7 (15.0)	68.1 (60.0,89.4)	55.6–98.8	70.7 (14.6)	73.1 (61.3,78.1)	44.4–98.8	23.5	0.68

RNL = Reintegration to Normal Living Index; ABC = Activities-specific Balance Confidence Scale. Participants with data in both seasons.

The activity monitor data in Table 5 includes data from the participants who had activity monitor data over both seasons. Median (25^th^,75^th^ percentile) steps per day was 7717.2 (5748.4,10420.8) in winter and 7003.0 (4373.6,13183.9) in summer, Wilcoxon Signed Rank Test Z = 32.0 (p value 0.65). The median number of longer walking bouts (>300 steps per bout) was 1.0 (0.3,2.3) in the winter and 0.9 (0,6.4) in the summer. Mean temperature over the wear time of the activity monitors for each season ranged from minus 9.0 degrees Celsius in the winter to 20.7 degrees Celsius in the summer.

**Table 5:** Activity monitor outcomes by season.

	Winter (n = 10)		Summer (n = 10)		Difference	
Measure	Mean (SD)	Median (25^th^,75^th^ percentile)	Mean (SD)	Median (25^th^,75^th^ percentile)	Wilcoxon Signed Rank Test Z	p value
**Steps per day**	8325.1 (3568.7)	7717.2 (5748.4,10420.8)	8425.4 (5401.7)	7003.0 (4373.6,13183.9)	32.0	0.65
**Peak cadence per day**	86.8 (25.5)	81.1 (62.3,108.8)	80.9 (27.5)	68.6 (59.2,110.4)	15.0	0.20
**Peak 30 min cadence per day**	64.7 (26.7)	55.9 (43.5,86.7)	64.9 (26.4)	52.5 (43.2,90.6)	24.0	0.71
**# Bouts <40 steps, per day**	56.9 (22.4)	51.0 (37.7,78.0)	55.1 (16.2)	55.3 (42.7,71.0)	23.0	0.65
**# Bouts 41–299 steps, per day**	35.1 (18.9)	30.5 (20.7,48.9)	38.9 (19.9)	34.2 (22.0,55.2)	42.0	0.14
**# Bouts >300 steps, per day**	1.7 (2.0)	1.0 (0.3,2.3)	2.9 (4.1)	0.9 (0,6.4)	37.0	0.33
**Number of wear days**	6.8 (0.6)	7.0 (6.0,7.0)	6.5 (1.2)	6.5 (5.8,7.0)	12.0	0.37
**Mean temperature over wear time (degrees Celsius) [range]**	-9.0 (7.6) [-16.8 to 7.1]	-11.2 (-14.4,-3.5)	20.7 (1.6) [18.5 to 23.9]	20.9 (19.8, 21.5)	0.0	0.005

Participants with data in both seasons.

The STROBE checklist was completed. See S1 Checklist. S1 Data is anonymized and aggregated data from Table 4 and S2 Data is anonymized and aggregated data from Table 5.

### Qualitative

Eight participants agreed to be interviewed, six of whom were female. See Table 6 for characteristics of these participants. Five of those participants were interviewed in both summer and winter seasons and three during only one season for a total of 13 interviews. We reached data saturation with no additional new information attained and no further coding identified with the final interviews. To establish credibility, we completed member checking with participants. We provided a description of the study context to ensure transferability and developed an audit trail of the data analysis process for dependability. All of these processes were part of the decision-making trail that helped establish the study’s confirmability [41,43].

**Table 6:** Description of participants in qualitative component at initial assessment time.

	Frequency or Mean(SD)	Median (25^th^,75^th^ percentile)
**Season interviewed**	5 (B); 1 (W); 2 (S)	-
**Sex**	6 (F); 2(M)	-
**Age (years)**	57.8 (14.0)	58.5 (49.8,70.0)
**Time post stroke (weeks)**	336.3 (274.8) [6.5 years]	218.5 (112.8,618.25) [4.2 years]
**SIS recovery (0–100)**	74.4 (11.5)	75.0 (62.5, 86.3)
**RNL (0–33)**	27.6 (4.1)	28.0 (23.0, 31.0)
**TUG (seconds, lower score is better)**	22.4 (14.6)	20.8 (8.6, 37.0)
**ABC item ‘walk outside on icy sidewalks’ (0–100)**	31.3 (30.4)	25.0 (2.5,57.5)
**Steps per day (mean)**	9773.6 (5299.0)	9108.0 (5930.0,13979.0)

B = both seasons, W = winter, S = summer; SIS = Stroke Impact Scale; RNL = Return to Normal Living Index; TUG = Timed Up and Go; ABC = Activities-specific Balance Confidence Scale.

#### Themes

Participants described numerous factors that influenced participation when discussing their RNL scores; however, for the purpose of the data analysis, we focused on seasonal factors. Three themes were identified: 1) Feel more independent, get out more in the summer; 2) Weather conditions create restrictions; and 3) Active despite challenges of winter.

*Feel more independent*, *get out more in the summer*. This theme highlights the overall sentiment of participants related to seasonal participation. As one participant expressed, (ID 2 Summer, female) “I feel like I do less in the winter than I do in the summer.”. Participants talked about being less active in the winter, (ID 6 Summer, female) “…in wintertime, I can’t do half as much, my exercising and that sort of stuff.”. (ID 20 Summer, female) “Oh for sure. Winter, winter, um, I, I am, I’m not very active person in, in winter I’m not.”

Participants felt much more independent in the summer. (ID 2 Winter, female)“And in the summertime, we actually just go wherever we want.”

Participants found it easier to stay active even if they did not always feel up to it.

(ID 20 Winter, female) “Summer I’d be out every day. Every day… Even if I had a headache, you know, I wake up, I wake up with headaches and I’ll have them all day but that I’ll still, you know, ride my bike. I’ll still be active”.

*Weather conditions create restrictions*. Participants gave numerous examples of winter weather conditions that caused challenges, such as ice, snow, cold, and wind.

(ID 2 Summer, female) “I feel like I do less in the winter than I do in the summer because I’m in and because of the roads and the snow and, uh, I just feel like it’s, it’s way easier for a handicapped person to move around in the summertime when they don’t have the snow and the ice and, and the wind.”

These environmental challenges led to difficulties mobilizing on sidewalks, using walking aides and wheeled mobility in the snow and ice. People described getting stuck, being cold and being fearful of falling.

Mobility

(ID 7 Winter, female) “Mostly the ice because it’s very dangerous and with limited balance and having to have a walker or being in a wheelchair. Winter weather is just a real barrier.”

(ID 20 Summer, female) “And, and it’s a really, really bad in the suburbs. Really bad. And if there’s anything I would ask the city to improve is, is the sidewalk conditions in sum, in winter I mean.”

Fear of Falls

(ID 7 Winter, female) “And that means taking the risk of standing on ice or slipping. And if I fall, I’m unable to get up.”

(ID 11 Winter, female) “I am nervous of falls just because I’ve had them before… Ice makes me nervous when I’m out walking for sure.”

Restricted travel due to weather conditions was discussed as hesitancy to drive and not being able to go on a trip or out of town.

(ID 7 Summer, female) “Yea. I didn’t, last year I didn’t, uh, I didn’t have my driver’s license during winter months. And in the spring, I waited until all the snow was gone before I ventured out with the walker and the car by myself.”

Winter clothing required by cold weather caused some ADL limitations due to the bulkiness of the clothing restricting movement and increased time to get dressed.

(ID 6 Summer, female) “Because it restricts you in the car and once you put it, you’re going to get in a car, it restricts you now when you get in any place it restricts you. Like you’re, you’ve gotten twice as much clothing on you, you know, you feel it.”

Winter was also more emotionally challenging for many participants. Some participants described being depressed, associating their mood with winter weather.

(ID 20 Winter, female) “And I’m starting to feel it. I’m getting cabin fever and starting to get, you know, kind of down on myself and, you know, the whole depressing unworthy feeling.”

(ID 11 Winter, female) “Nothing I’m doing is within walking distance so I’m kind of just zipping around the city but, um, the weather definitely brings me down a little bit.”

Summer environmental challenges that people discussed included difficulty with rain, wind, and slipping while walking in sand.

(ID 7 Summer, female) “Should mention I’m kind of a coward when it comes to rain. If it’s rainy outside and I have an option, I might not go by myself.”

*Active despite challenges of winter*. Despite the various challenges that people described in the winter, six of the eight participants gave numerous examples of finding other ways to keep physically active, such as walking in a hallway, indoor skywalks or using a stationary bike.

(ID 2 Summer, female) “It probably affects me like quite a bit because if it’s lots of snow on the sidewalks, I don’t go out. I walk in, I vary, in my apartment or I walk in the hallway.”

For those who had social support available, they were able to participate and go out or see friends due to that assistance.

(ID 7 Winter, female) “My husband is very, very accommodating. For instance, tonight we will go to a concert. We go to many, many concerts and shows because we both like them and because music is very important to me and because my family is involved. So summer and winter, we do that a lot.”

Participants also described doing things anyway despite challenges: the winter weather led to activities taking longer to complete, such as walking a specific distance outdoors, activities were more difficult to complete in winter weather.

(ID 11 Winter, female) “Well activities that I do now, um, definitely going to the gym. Like that is fatiguing, especially walking there and back in the freezing cold. When it’s nicer out it’s not so bad. Um. Also just running lots of errands.”

## Discussion

For the first objective, differences in participation and activity were not noted between seasons with the quantitative data. Interpreting the quantitative and qualitative results together was very important in this study, to further explain these findings. For example, participants frequently discussed the challenges with walking in ice and snow. This is reflected in the ABC score for the item regarding confidence to ‘walk outside on icy sidewalks’, which had a mean score of 31.3 (median score of 25.0) for the eight participants included in the qualitative component, with a mean age of 57.8. This compares to a mean score of 41.7 on this item in a sample of 77 stroke survivors on average four years post stroke [25]. In a study of people at one year post stroke, the mean total ABC score was 73.24, similar to the participants in this study [44].

Participants described concerns with the potential for falling outside in the winter. The TUG score average of 22.4 seconds for the qualitative participants suggests being at risk for falls [34]. This is a higher (worse) score than noted in a study of stroke survivors at one year post stroke in which the mean TUG score was 15.80 seconds [44]. The high TUG score in this study means that it takes longer to complete the TUG tasks. The TUG score in combination with low confidence in walking on ice helps to illuminate some of the concerns regarding falling in this group. In a Japanese study of community dwelling stroke survivors, falls did occur more frequently in winter than the other seasons [11].

The average temperature difference between assessment seasons was 29.7 degrees Celsius. Other studies have found less physical activity and participation in Canadian adults (aged 19 plus) in winter compared to summer [45,46]. We did not note apparent differences in participation and activity (RNL and activity monitor data) between seasons, though the large extremes of temperature in the city where this study occurred were not observed during the study period. In the winter months, the temperature extremes can be -40 degrees Celsius or lower with windchill, and in the summer months 35 degrees Celsius or higher.

Although people described feeling more independent and getting out more in summer when interviewed, they also described many ways of participating and being active in the winter despite the difficulty. Of the eight people interviewed, six people described participating and being active despite winter challenges and found alternatives, learning to adapt to winter. This finding helped to explain the estimated lack of difference in the measures of activity and participation between seasons. A similar finding was seen in a sample of 502 adults from the USA with a mean age of 54. The authors found that even when weather conditions were icy most participants continued to participate in work and volunteer activities. They continued to go out, despite the challenges [47]. A study from Calgary, Canada, found that for 912 participants aged 60+, recreational walking was more likely to occur in spring relative to winter [48]. No seasonal differences relative to winter were identified for walking for transportation purposes, moderate intensity physical activity or vigorous intensity physical activity, similar to our activity monitor findings with community dwelling people with stroke [48].

Social supports enabled some participants to remain physically active indoors and to participate in social activities despite the challenges weather conditions presented. It has been noted that after stroke, social support is important to maintain or increase participation [49]. In another study, older adults were able to continue meaningful physical activity as they aged when they had support to adapt to things which may limit their activity [50]. It has been suggested that no matter what sort of environmental barriers may be present after stroke, people may be able to engage in social participation when they have appropriate social support [51]. The availability of social supports would be an important area to explore with individuals post-stroke to facilitate activities and participation. Other factors such as socio-economic status and gender were not evaluated in this study and should be further explored.

Some participants who were interviewed described a depressed mood, associating it with winter weather. Post-stroke depression has been noted to be higher in winter than in summer, with the association mediated by vitamin D level [52]. Those with a depressed mood are also less likely to participate in physical activities [53]. Consequently, depressed mood should also be considered when examining seasonal participation outcomes post-stroke.

The participants in this study were active, motivated members of a stroke recovery association and were, on average, six years post stroke. Only three (23%) of participants described using a walking aid. In a Scottish study evaluating the physical activity of 22 people on average four years post stroke, the average steps per day was 4035 [6]. This is less than half of the average of 8425 steps per day in summer with the participants in this study. The two studies used different activity monitors in different locations and with different analyses. This study had the monitor attached to the ankle of the less-affected side and used an LFE analysis which is more sensitive when people walk slowly–both of which could lead to higher step counts [54]. While ActiLife’s LFE filter has been shown to be accurate for use in individuals with gait speeds < 1.0m/s [26,27,55], many researchers do not have participants wear the monitor at the ankle. Although the monitor is more sensitive in detecting steps in people who walk slowly when worn at the ankle [26], it may also erroneously record other movements (e.g., fidgeting) as steps.

It has been previously noted that in people with stroke, the largest number of bouts per day is in the short bout category (<40 steps), with the number of long bouts (>300 steps) being the smallest category [30]. In this study, the largest number of walking bouts per day were also in the short bout category for both seasons. The number of long bouts per day was much smaller than the number of short and medium bouts. The number of short and medium bouts in each season appeared similar, with the mean number of longer bouts 1.7 times greater in the summer, however, median scores did not appear to differ. Statistical analysis of differences between seasons was carried out with the Wilcoxon Signed Rank Test due to small sample size. This should be further studied with a larger sample size in future.

There were a number of limitations in this study. Recruitment was slow and that led to a smaller than ideal sample size. Participants started the study in different seasons. There are potentially other self-perceived and observed outcome measures which may be more suited to studying seasonal differences than the measures which we chose to use. Based on our findings and experience with recruitment and outcome measures used in this study, we have made a number of suggested changes for future seasonal studies (see Table 7).

**Table 7:** Future suggestions.

Challenge	Potential change
**Recruitment**	Additional recruitment strategies such as through inpatient rehabilitation settings, in outpatient rehabilitation departments, home therapy services, community recreation services for seniors and individuals with disabilities, and specialist offices (such as physiatrists, family medicine and neurology).
**Inclusion criteria**	Include participants less than 12 months post stroke and those who are not ambulatory.
**Outcomes**	
Participation	For the participation outcome, a measure such as the Community Health Activities Model Program for Seniors (CHAMPS) could identify hours of meaningful activities in an average week [56]. This measure has been used as a measure of participation in previous studies with community dwelling people with stroke [24].
Activity	Moderate and vigorous physical activity can be estimated from the CHAMPS [57]. Items related to outdoor walking can also be identified [58] Outdoor physical activity could also be evaluated by additionally using GPS with an activity monitor [59]. Larger studies or situations where an activity monitor is not possible may benefit from using a self-report measure of physical activity and outdoor walking. We chose outcomes considered to be less invasive and able to be completed in a person’s home. Future studies may benefit from the use of other common measures of activity such as a measure of balance, for example, the Berg Balance Scale [60] or mini-BESTest [61]; a measure of endurance, for instance, the Six-Minute Walk Test (6MWT), or gait speed.[62,63]

Future research questions include the following: For those who are physically active or not active in summer months, is there a difference in activity and participation across seasons?; If a person was active prior to a stroke, are they active in any season after stroke?; Is there a difference in summer and winter activity and participation when less than 12 months post stroke and longer than 12 months post stroke?; Are there seasonal differences in activity and participation of individuals after stroke who are not ambulatory?; How do we encourage and assist safe activity and participation in winter? How important is social support to seasonal participation post stroke?

## Conclusion

Using mixed methods was very important in this study: the qualitative component results helped the authors to understand and put into perspective the quantitative findings. Participants described many challenges with winter weather, but also ways they had discovered to participate and be active despite these challenges.

Results may not be generalizable to all community dwelling individuals with stroke. However, results of this study may assist rehabilitation clinicians in determining appropriate goal directed and client focused intervention strategies for community dwelling stroke survivors. Listening to what seasonal challenges exist for each client and what adaptations they have been able to make will assist in addressing these challenges.

Changes to inclusion criteria and participation outcomes for future studies into seasonal differences of participation and activity after stroke are suggested. There are still many questions regarding seasonal differences and challenges to both activity and participation in winter and summer for stroke survivors. Potentially, this will lead to a focus on winter specific interventions aimed at making seasonal participation and activity less challenging for people with stroke.

## Supporting information

S1 Checklist STROBE statement—checklist of items that should be included in reports of cohort studies. (DOCX)

S1 Data Aggregated data ABC and RNL. (XLSX)

S2 Data Aggregated data activity monitor. (XLSX)
